# Electroluminescence properties of InGaN/GaN multiple quantum well-based LEDs with different indium contents and different well widths

**DOI:** 10.1038/s41598-017-15561-9

**Published:** 2017-11-10

**Authors:** Changfu Li, Ziwu Ji, Jianfei Li, Mingsheng Xu, Hongdi Xiao, Xiangang Xu

**Affiliations:** 1 School of Microelectronics, Shandong University, Jinan, 250100 China; 2 School of Physics and Electronic Engineering, Taishan University, Taian, 271000 China; 3 State Key Laboratory of Crystal Materials, Shandong University, Jinan, 250100 China

## Abstract

Two InGaN/GaN multiple quantum well (MQW)-based blue light emitting diodes (LEDs) emitting photons at approximately the same wavelength, with different indium contents and well widths, are prepared, and the temperature-dependences of their electroluminescence (EL) spectra at different fixed injection currents are investigated. The results show that, compared with sample B with its lower indium content and larger well width, sample A with its higher indium content and smaller well width, has a stronger carrier localization effect and higher external quantum efficiency (EQE) at the lower fixed currents; however, upon increasing the injection current, both the localization effect and EQE for sample A decrease at a faster rate. The former is mainly attributed to the deeper potential levels due to the larger indium fluctuations originating from the higher indium content, and to the smaller well width-induced stronger carrier quantum-confine effect (QCE); the latter is mainly attributed to the more significant growing in the electron leakage and/or electron overflow originating from the smaller well width and larger lattice mismatch-induced stronger piezoelectric field, and to the more significant reduction in carrier localization effect originating from the smaller well width-induced smaller density of high-energy localized states.

## Introduction

InGaN/GaN multiple quantum wells (MQWs) acting as active layers in light emitting diodes (LEDs) and laser diodes (LDs) have attracted significant attention, since by tuning the indium composition in an InGaN well layer, the whole spectral range, corresponding to near-infrared to visible and up to near-ultraviolet emissions, can be covered by the nitride system^1–4^. InGaN/GaN MQW-based blue LEDs have been extensively employed in full-color displays, back-lighting, general illumination, and in other applications of optoelectronic devices, at the same time, green, yellow, and even red long-wavelength InGaN/GaN MQW-based LEDs that are used when preparing white LEDs, are attracting much research interest; however, due to the large discrepancy in atomic size between indium and gallium, and a large lattice mismatch of 11% between InN and GaN, either a phase separation or a slight composition fluctuation always occurs in the InGaN well layers, and this also results in generation of the structural defects acting as non-radiative recombination centers. Although the former is advantageous in improving the radiative recombination efficiency due to the carrier localization effect, the latter is detrimental to the radiative recombination efficiency because of enhancement of non-radiative recombination. Also, due to a large lattice and thermal mismatch between the InGaN well layer and the GaN barrier layer, a large strain is induced in the MQWs, which can generate a large piezoelectric field in the MQW region. This induces a quantum-confined Stark effect (QCSE) therein, and therefore reducing the radiative recombination efficiency of the carriers in the MQWs^5–10^. Moreover, the piezoelectric field also facilitates electron leakage, or electron overflow, from the MQW layers to the deep levels in the GaN barriers or p-GaN layer, thus leading to a further reduction of the radiative recombination efficiency^11^. Therefore, to produce a high-efficiency InGaN/GaN MQW-based LED, the indium content must be reduced, however, to produce a high-efficiency InGaN/GaN MQW-based LED with a fixed emission wavelength, a reduction in the indium content is bound to require an increase in well width to maintain a constant emission wavelength. Nevertheless, the increase in the well width, in turn, results in a decrease of the radiative recombination efficiency of the carriers in the MQWs due to the increase of the spatial separation of electrons and holes and the reduction of the quantum-confine effect (QCE) of the carriers, although it also simultaneously suppresses the electron leakage or electron overflow due to the reduction of the carrier QCE^12–14^. Therefore, for the InGaN/GaN MQW-based LED with a fixed emission wavelength, its resulting comprehensive optical performance, in fact, is mainly a result of the competition between the indium content and well width. Consequently, to obtain such a high-efficiency LED with a fixed emission wavelength, it is necessary to investigate the combined effects of the indium content and well width on the optical properties of LED.

In this research, to produce a high-efficiency blue LED, two InGaN/GaN MQW-based LEDs, with different indium contents and different well widths, were grown, and the temperature-dependences of their electroluminescence (EL) spectra at different fixed injection currents are investigated to explore the effect of the indium content and well width on the carrier transferring and recombining mechanism in such LEDs.

## Results and Discussion

**Figure 1:**
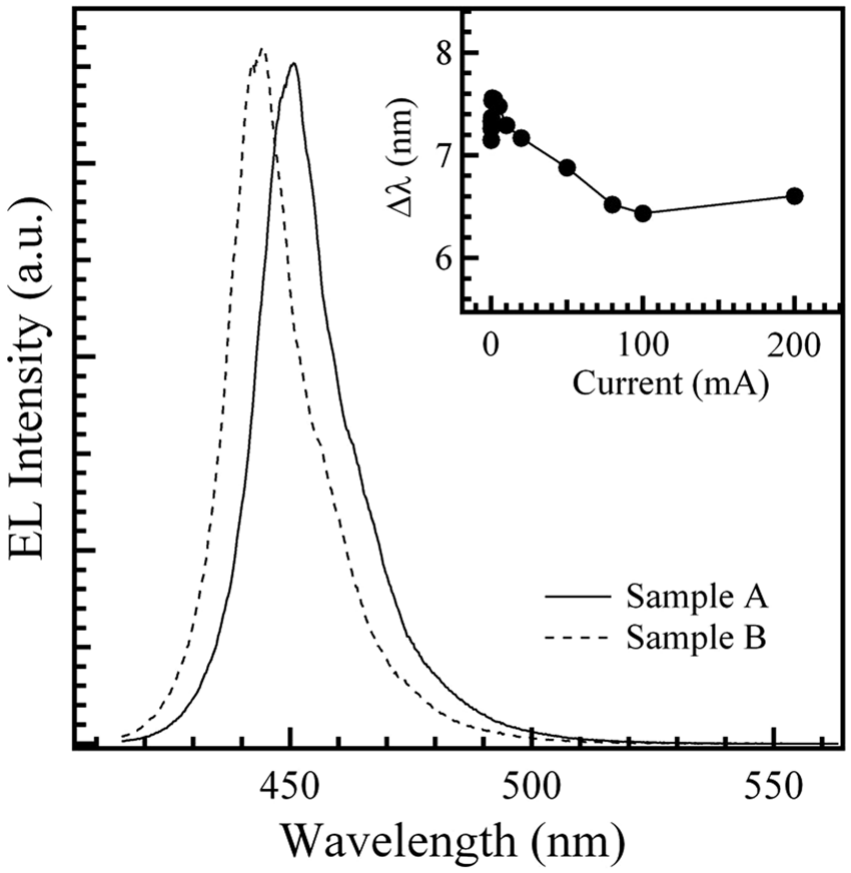
200 mA EL spectra of samples A and B at 300 K. The inset shows the wavelength difference (Δ*λ*) between samples A and B as a function of the injection current at 300 K.

**Figure 2:**
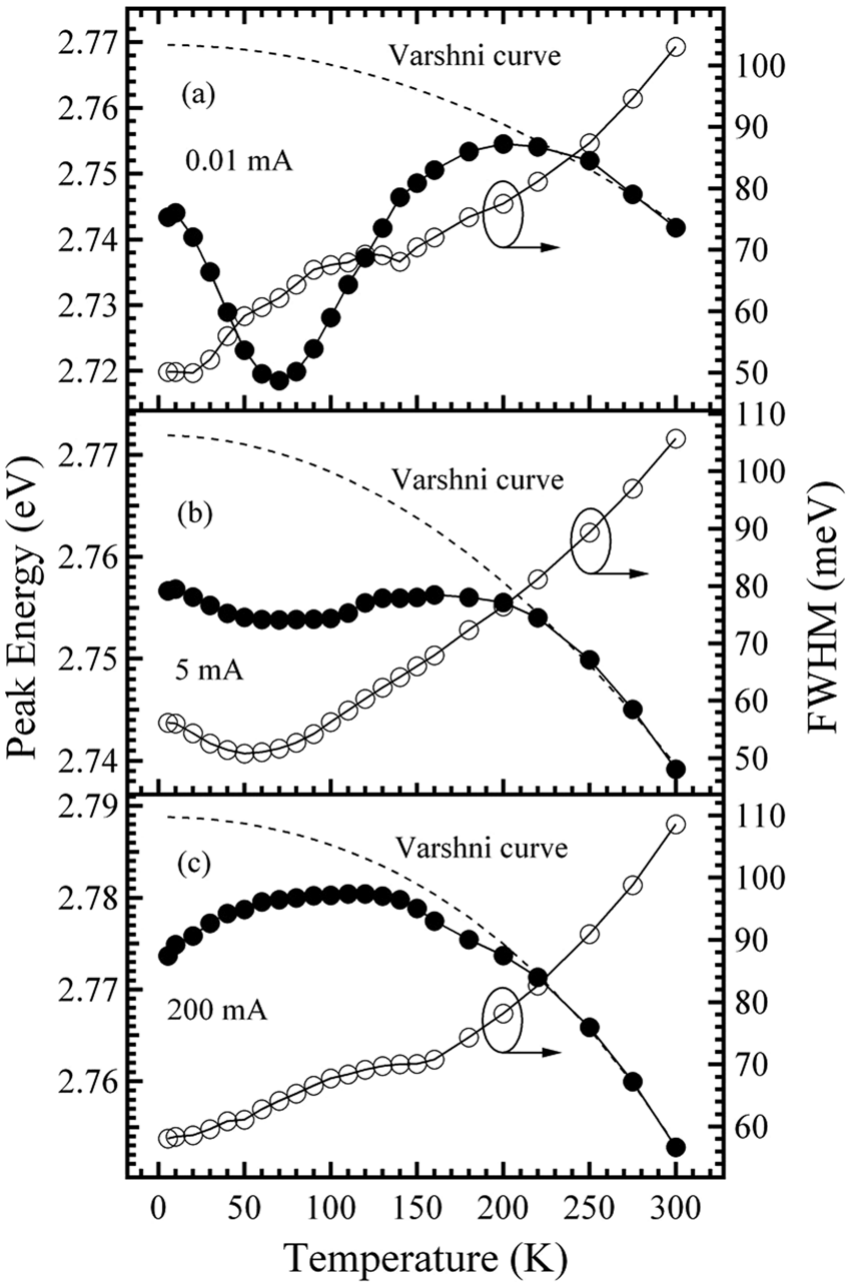
Temperature dependences of the peak energy and full width at half-maximum (FWHM) for sample A measured at 0.01 (**a**), 5 (**b**), and 200 mA (**c**). The dashed lines represent Varshni curves.

**Figure 3:**
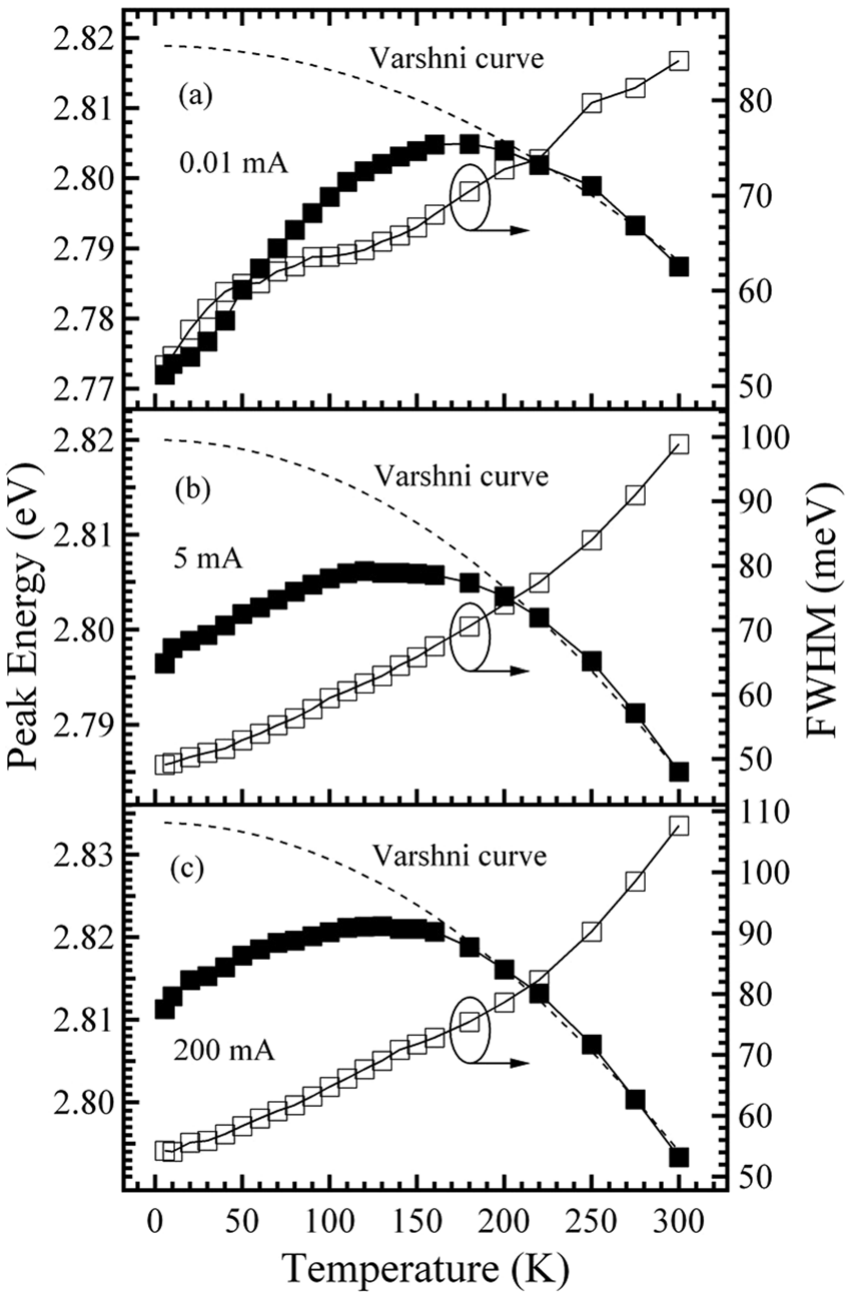
Temperature-dependences of the peak energy and FWHM for sample B measured at 0.01 (**a**), 5 (**b**), and 200 mA (**c**). The dashed lines represent Varshni curves.

Figure 1 shows EL spectra of samples A and B measured at 300 K and 200 mA. Both of the EL spectra exhibit one InGaN-related main emission peak. The slight hump on the long-wavelength side of the main EL peak represents phonon replicas of the main peak, because the peak energy of the hump is about 90 meV lower than that of the main EL peak. The inset in Fig. 1 shows the wavelength difference (Δλ) between samples A and B as a function of the injection current at 300 K. As can be seen from the inset, the Δλ value is no more than about 7 nm, especially at higher injection currents, indicating that the two InGaN/GaN MQW-based LEDs, with varying well widths and different indium contents, have approximately the same emission wavelength, as expected. To investigate the effect of the indium content and well width on the carrier transfer and recombination mechanism of the LED structure, Figs 2 and 3 illustrate the temperature-dependencies of the EL peak energy and linewidth of the two samples A and B recorded at the typical fixed injection currents of 0.01, 5, and 200 mA, respectively.

First, for sample A, as shown in Fig. 2(a), the anomalous temperature behavior of the EL spectra at the fixed injection current of 0.01 mA is seen to be “S-shaped” (decrease-increase-decrease) for the peak position and “W-shaped” (decrease-increase-decrease-increase) for the peak linewidth, which was attributed to the potential inhomogeneity, and localized nature, of the carrier recombination due to composition fluctuations in the InGaN matrix, however, upon increasing the fixed injection current from 0.01 to 5, and further to 200, mA, the temperature behavior of the peak energy (linewidth) gradually evolves from a strong “S-shaped” (“W-shaped”) temperature-dependence into a weak “S-shaped” (an approximately “V-shaped”) relationship, until becoming an inverted “V-shaped” (a monotonically increasing) temperature-dependence, indicating that, upon increasing the injection current, the carrier localization effect gradually reduces. A similar phenomenon has also been observed in photoluminescence (PL) measurements using a similar blue InGaN/GaN MQWs structure^7,15^.

On the other hand, for sample B, it is found from Fig. 3 that, differing from the case of sample A shown in Fig. 2, regardless of the fixed injection current (within the selected measurement range of this work), the anomalous temperature behavior of the peak energy (linewidth) exhibits an inverted “V-shaped” (monotonic increase). That is, the “S-shaped” temperature-dependence of the peak energy accompanied by the “W-shaped” or “V-shaped” temperature-dependence of the linewidth, as shown in Fig. 2(a) and (b) for sample A, is not observed for sample B. The behavior seems to indicate that the MQWs of sample B should have a better homogeneity in the depths of the localized states than that of sample A owing to the lower indium content, thus leading to the absence of the temperature-dependent relaxation process of the carriers in the MQWs from shallower down to deeper localized states by hopping.

**Figure 4:**
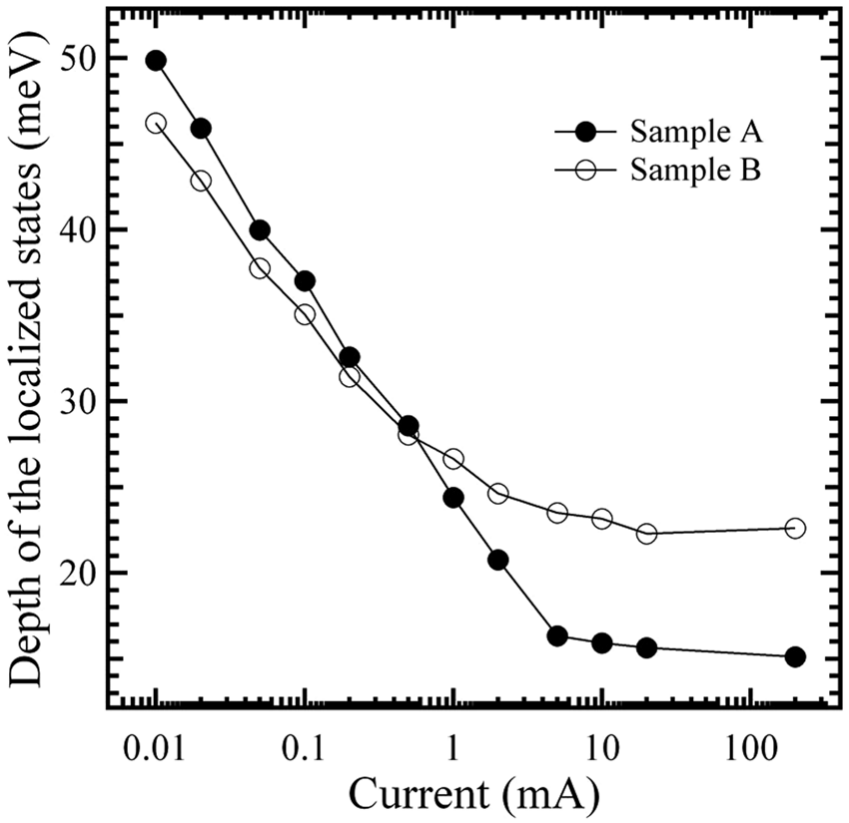
Depth of the localized states as a function of injection current for samples A and B.

To investigate and compare the extent of carrier localization of the two MQWs, the depths of the localized states for the two samples are estimated by using Varshni’s equation to fit the peak energy *v*. temperature curves at different fixed currents in the injection current range of 0.01–200 mA (Figs 2 and 3)^16^. The values of the depth of the localized states obtained at various injection currents for the two samples, as a function of the injection current, are plotted in Fig. 4. As can be seen from Fig. 4, upon increasing the fixed current from 0.01 to 200 mA, both samples show a decrease in the depth of the localized states, a result in good agreement with the fact that, with an increase in the injected carrier concentration, the carrier localization effect gradually decreases^17^. Moreover, it is also found from Fig. 4 that, compared with sample B, the depth of the localized states for sample A is larger in the lower current range below the critical current of about 0.5 mA, and smaller in the higher current range above about 0.5 mA, indicating that compared with the former, the latter has a stronger localization effect in the lower current range, and a weaker localization effect in the higher current range.

**Figure 5:**
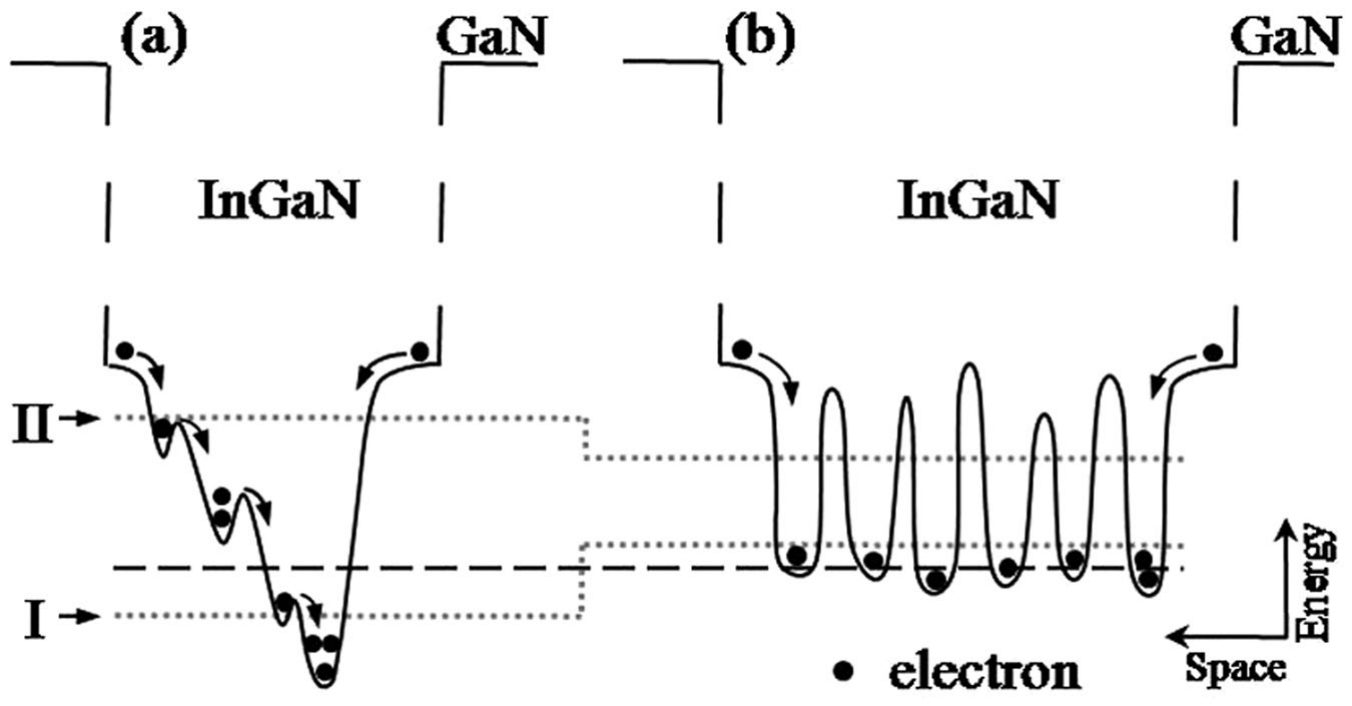
Schematic diagrams indicating the possible state of carriers distributing in the MQW structure for samples A (**a**) and B (**b**). The dashed line indicates the lowest energy level of the localized states in sample B. Dotted lines I and II indicate the highest energies of the localized states filled by the injected carriers in the lower and higher injection currents, respectively.

To explain the aforementioned injection current-dependent behavior with regard to the localization effect, the schematic diagrams indicating the possible states of carrier distributing in the MQWs of the two samples are shown in Fig. 5. To facilitate comparison of localization effect, the bottoms of the InGaN conduction band for the two samples are set at the same height in the energy. For sample A, as can be seen from Fig. 5(a), the density of states in the band tail, which exponentially increases with energy up to the free-exciton energy, is taken into account; in contrast, for sample B, the density of the localized states is considered to be slight energy-dependent or approximately energy-independent up to the free-exciton energy (Fig. 5(b)). Also, Fig. 5 shows that, compared with sample B, the depths of the lowest-energy localized states below the dashed line for sample A are deeper. Thus, at the lowest current of 0.01 mA and at the lowest temperature of 6 K, the highest energy level of the localized states filled by the injected carriers for sample A, should be lower than that for sample B, as marked by the dotted line I in Fig. 5. That is, the lowest-energy localized states below the dashed line for sample A are only partially filled in this case. Therefore, for sample A, upon increasing the temperature from 6 K in the initial temperature range, the weakly localized carriers are thermally activated and relax downwards into strongly localized states by hopping and reach a saturated redistribution, which results in the initial red-shift of the peak energy accompanied by the initial decrease of the peak linewidth, in good agreement with that observed in Fig. 2(a) ^18,19^. In contrast, for sample B, due to the better homogeneity of its localized state depths, the increasing temperature in the initial temperature range can enable the localized carriers to reach thermal equilibrium with the lattice and to occupy higher-energy levels of the localized states, thus leading to an initial blue-shift of the peak energy towards the free-exciton ground states accompanied by the initial increase of the peak linewidth, in good agreement with that observed in Fig. 3(a). The aforementioned analysis based on the energy band model shown in Fig. 5, explains the experimental results obtained in Figs 2–4 which showed that sample A has a more significant localization effect than sample B at the lowest injection current of 0.01 mA; however, at 6 K, and when the fixed current is further increased to some current above the critical current of 0.5 mA, such as to 5 or 200 mA, for sample A, besides the fact that the lowest-energy localized states below the dashed line are fully filled due to its smaller density of localized states, the higher-energy localized states above the dashed line are also partially filled by the injected carriers. For a certain higher energy level above the dashed line, however, since the density of the localized states for sample A is smaller than that in sample B due to the smaller well width, the highest energy level of the localized states filled by the injected carriers for sample A may be higher than that for sample B at the higher currents, as marked by the dotted line II shown in Fig. 5. Therefore, compared with sample B, sample A should have a less significant localization effect in the higher current range above the critical current of 0.5 mA, in good agreement with the results obtained in Figs 2–4.

**Figure 6:**
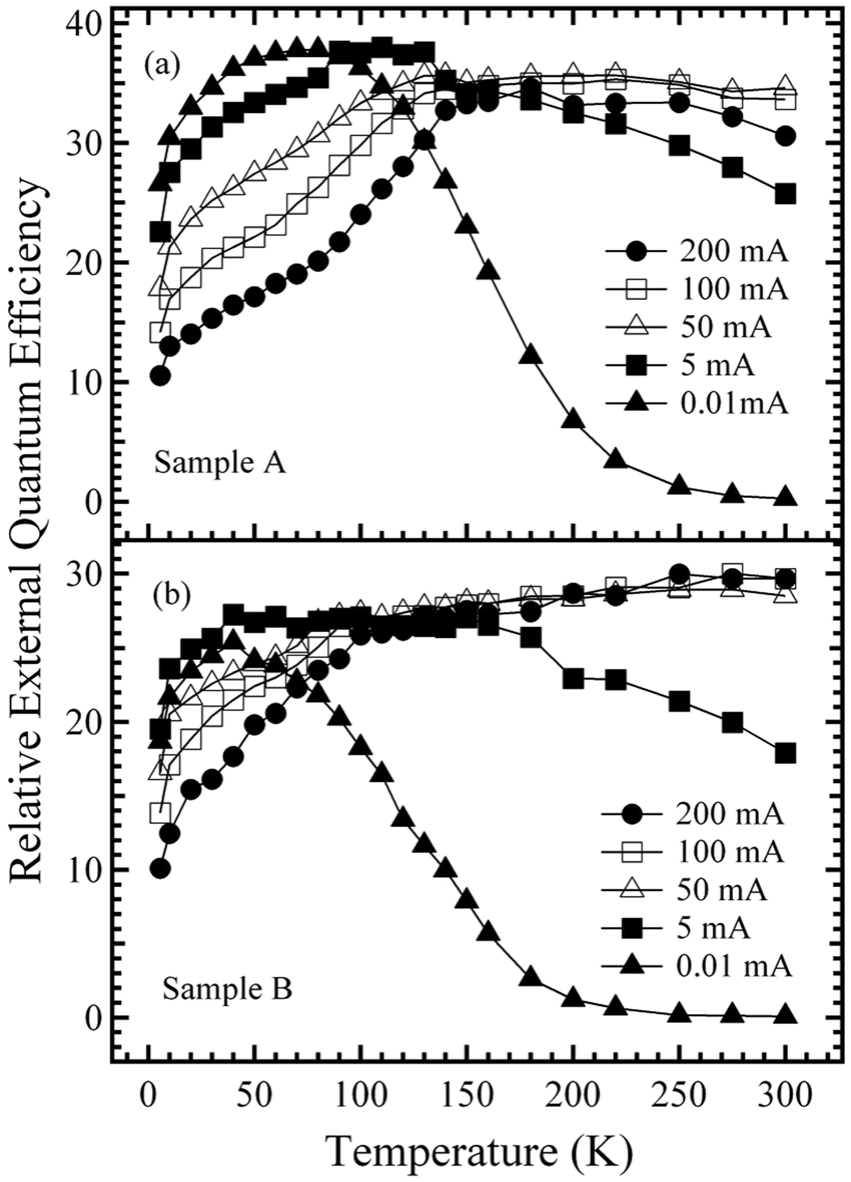
Temperature-dependence of relative external quantum efficiency (EQE) for samples A (**a**) and B (**b**), measured at 0.01, 5, 50, 100, and 200 mA.

Moreover, to investigate the effect of the indium content and well width on efficiency, the integrated EL intensity divided by the current, *i*.*e*., relative external quantum efficiency (EQE), is plotted as a function of temperature at the typical fixed injection currents of 0.01, 5, 50, 100, and 200 mA for the two samples (Fig. 6). For sample A, as shown in Fig. 6(a), when the current is fixed at 0.01 mA and the temperature is lowered from 300 to 6 K, the EQE value increases initially reaching a maximum at the critical temperature of about *T*_A0_ = 70 K, and then decreases. The former can be explained mainly by deactivation of the non-radiative recombination centers, which are thermally active at 300 K, the latter is mainly attributed to the growing electron leakage resulting from the increasing forward bias^20–23^. When the fixed current is increased from 0.01 to 200 mA, the trend, which the EQE value first increases and then decreases with lowering temperature, remains unchanged, however, it is found that in the higher temperature range, upon increasing the fixed current from 0.01 to 50 mA the increasing of the temperature-dependent EQE value becomes less and less significant, and while the EQE curve gradually shifts to a higher value overall. The behaviors can be mainly attributed to the gradual saturation of the non-radiative recombination centers upon increasing the fixed current. Nevertheless, when further increasing the fixed current to above 50 mA, the increase of the temperature-dependent EQE value becomes more significant, and while the EQE curve shifts to a lower value overall, compared with the case at 50 mA, indicating that, when increasing the fixed current from 50 mA to 200 mA the electron overflow at the higher temperatures gradually increases^24–26^. In contrast, in the lower temperature range and when the fixed current is further raised from 0.01 to 200 mA, the decrease in the temperature-dependent EQE value becomes more significant, and while the EQE curve shifts to a lower value overall. This is mainly attributed to the growing electron leakage resulting from the increasing forward bias.

On the other hand, for sample B, it is found from Fig. 6(b) that in the lower fixed current range below about 50 mA, the temperature behavior of the EQE value, and the current behavior of the EQE curve, are similar to those observed in the lower fixed current range in Fig. 6(a) for sample A; however, at the higher fixed currents of 50 mA, and above, the temperature behavior of the EQE value for each fixed current is almost monotonically reduced over the whole temperature range of 300–6 K. This indicates that, differing from the case of sample A shown in Fig. 6(a), for sample B, the temperature behavior of the EQE value in the higher temperature range and higher current range, remains dominated by electron leakage rather than electron overflow. This is also in good agreement with the experimental data shown in Fig. 6(b) within the higher temperature range: when the fixed current is further increased from 50 to 200 mA, the shift of the EQE curve to a lower value overall as shown in Fig. 6(a) for sample A, is not observed for sample B. Furthermore, for sample B, the absence of electron overflow within the higher temperature range, and higher current range, is mainly attributed to its larger well width and weaker piezoelectric field originating from the lower indium content^27–29^.

Moreover, it is also found from Fig. 6 that, at the fixed current of 0.01 mA, the EQE curve of sample A is significantly higher than that of sample B overall. This may be mainly attributed to the aforementioned fact that sample A has a stronger carrier localization effect and a larger wave function overlap between the electron and hole originating from its smaller well width-induced stronger carrier QCE (Figs 2–4)^30^. However, when the fixed current is increased from 0.01 to 200 mA, the EQE curve of sample A gradually decreases at a faster rate compared with that of sample B. This behavior may be mainly attributed to the aforementioned fact that sample A has a more significant growth in electron leakage and/or electron overflow, due to its smaller well width and larger lattice mismatch-induced stronger piezoelectric field and its more significant reduction in carrier localization effect originating from the smaller well width-induced smaller density of high-energy localized states (Figs 2–4).

## Conclusions

In summary, to produce high-performance InGaN-based LEDs that emit photons at a fixed wavelength, two blue InGaN/GaN MQW-based LED samples, with different indium contents and well widths, have been grown, and the temperature-dependences of their EL spectra investigated at different injection currents. The results show that, compared with sample B with its lower indium content and larger well width, sample A with its higher indium content and smaller well width, shows a stronger carrier localization effect at lower currents due to its larger indium fluctuations originating from the higher indium content, but a weaker carrier localization effect at the higher currents due to its smaller density of high-energy localized states originating from the smaller well width. In addition, compared with sample B, the EQE *v*. temperature curve for sample A is higher with regard to the EQE value at the lowest injection current of 0.01 mA, and gradually decreases at a faster rate upon increasing the injection current from 0.01 mA: the former is mainly due to its stronger carrier localization effect mentioned above, and to its larger wave function overlap between the electron and hole originating from the smaller well width-induced stronger carrier QCE; the latter is mainly attributed to its more significant growth in the electron leakage and/or electron overflow due to the smaller well width and larger lattice mismatch-induced stronger piezoelectric field, and to its more significant reduction in the carrier localization effect originating from the smaller well width-induced smaller density of high-energy localized states. All of the experimental results show that the resulting comprehensive optical performance of the MQW structures with a fixed emission wavelength, in fact, is mainly a result of the competition between the indium content and well width as well as the injection current, and consequently exploring a suitable indium content and well width is crucial to improving the optical performance of such LEDs.

## Methods

### Sample fabrication

Two different InGaN/GaN MQW-based blue LED chips were grown on a (0001)-oriented sapphire substrate by using metal-organic chemical vapor deposition (MOCVD). The growth process is briefly described as follows: firstly a 25-nm-thick GaN buffer layer, followed by a 3-μm-thick unintentionally doped and a 3-μm-thick Si doping GaN layers are deposited on the substrate, then ten-period InGaN/GaN MQWs followed by a 20-nm-thick Mg-doped p-AlGaN electron blocking layer (EBL) and a 200-nm-thick Mg-doped p-GaN contact layer are grown on the GaN epilayers. For the InGaN/GaN MQWs, the thickness of the InGaN well layer is 2.6 nm for sample A with nominal indium contents of about 15.0% and 3.4 nm for sample B with its nominal indium content of about 12.8%. The LED chips, measuring 1.16 mm square, were fabricated using a conventional mesa structure method.

### Measurements

For temperature-dependent EL measurements at different fixed injection currents, the chips were mounted on a Cu cold-stage in a temperature-variable closed-cycle He cryostat to vary the sample temperature over the range of 6–300 K. A Keithley 2400 source meter was employed as an excitation current source over the current range of 0.01–200 mA. The EL signals from the samples were dispersed by a Jobin-Yvon iHR320 monochromator and detected by a thermoelectrical cooled Synapse CCD detector.
